# Development of a duplex SYBR Green I-based quantitative real-time PCR assay for the rapid differentiation of goose and Muscovy duck parvoviruses

**DOI:** 10.1186/s12985-018-1111-7

**Published:** 2019-01-10

**Authors:** Su Lin, Shao Wang, Xiaoxia Cheng, Shifeng Xiao, Xiuqin Chen, Shilong Chen, Shaoying Chen, Fusong Yu

**Affiliations:** 1 Institute of Animal Husbandry and Veterinary Medicine, Fujian Academy of Agriculture Sciences, Fuzhou, 350003 China; 2 Institute of Biotechnology, Fujian Academy of Agricultural Sciences, Fuzhou, 350003 China; 3 Fujian Animal Diseases Control Technology Development Center, Fuzhou, 350013 China

**Keywords:** GPV, MDPV, SYBR Green I, Duplex real-time PCR, Parvovirus

## Abstract

**Background:**

Waterfowl parvoviruses, including goose parvovirus (GPV) and Muscovy duck parvovirus (MDPV), can cause seriously diseases in geese and ducks. Developing a fast and precise diagnosis assay for these two parvoviruses is particularly important.

**Results:**

A duplex SYBR Green I-based quantitative real-time PCR assay was developed for the simultaneous detection and differentiation of GPV and MDPV. The assay yielded melting curves with specific single peak (Tm = 87.3 ± 0.26 °C or Tm = 85.4 ± 0.23 °C) when GPV or MDPV was evaluated, respectively. When both parvoviruses were assessed in one reaction, melting curves with specific double peaks were yielded.

**Conclusion:**

This duplex quantitative RT-PCR can be used to rapid identify of GPV and MDPV in field cases and artificial trials, which make it a powerful tool for diagnosing, preventing and controlling waterfowl parvovirus infections.

## Main text

Waterfowl parvoviruses cause substantial economic loss to waterfowl production worldwide [9, 12]. Parvoviruses that affect waterfowl are goose parvovirus (GPV) and Muscovy duck parvovirus (MDPV) [6, 31], both of which are members of the *Dependovirus* genus and *Parvoviridae* family. GPV and MDPV have linear single-stranded DNA genomes that are approximately 5 kb. The genome contains two large non-overlapping open reading frames (ORFs) flanked by two inverted terminal repeats (ITRs) at the 5′- and 3′- ends. The left ORF is predicted to encode a nonstructural protein NS, and the right ORF encodes the capsid proteins VP1, VP2 and VP3, which are derived from the same gene and differ in their N-terminal domain [7, 30].

GPV and MDPV possess approximately 77% homology in the VP1 nucleotide sequences or 85% at the protein level, and have been shown to be antigenically related to each other [7, 22, 31]. However, both parvoviruses have different hosts: GPV primarily infects geese and ducks, while MDPV infects ducks only [3, 9, 14, 20]. The classical method used to detect GPV and MDPV includes virus isolation and serological assays like seroneutralisation test (SN), latex particle agglutination test (LPA) and indirect immunofluorescence assays (IFA) [2, 10, 21, 33]. Despite of their precision and reliability, these methods are labor-intensive and time consuming. On the other hand, conventional polymerase chain reaction (PCR) and loop-mediated isothermal amplification assay (LAMP) [13, 15] were applied for fast identification of both viruses, but they do not quantify viral load, an important factor to measure for pathogenic and epidemiological studies.

Quantification method to detect waterfowl parvoviruses was previously reported using a TaqMan-based real-time PCR reaction containing primers targeting the parvovirus, as well as a probe labeled with fluorescent reporter dyes. However, these assays were not developed to differentiate GPV and MDPV [11, 16, 24, 28]. The method relying on a SYBR Green fluorescent quantitative PCR was also applied for detection and quantification of other viruses [23, 29]. Furthermore, it has been used to distinguish different virus DNAs in one reaction based on the peaks in the melting curves resulting from the difference in the *T*m values of the PCR products [1, 32]. Here, we developed a duplex SYBR Green-I based quantitative real-time PCR to rapidly and specifically amplify a region of the GPV and MDPV genome that can be used to distinguish infection by either virus in a single reaction.

Complete genome sequences of GPV strains (GenBank Accession No. HQ891825, JF926695 and KU844283) and MDPV strains (GenBank Accession No. NC_006147 and KU844281) [4–6] were aligned using DNAMAN Version 8 (DNASTAR, Madison, WI, USA). The alignment was used to identify conserved and diverged regions between the two parvoviruses. Based on the alignment, two pair of oligonucleotides SYBR Green primers specific to GPVs and MDPVs were designed in the VP1 gene using Primer Premier version 5 (PREMIER Biosoft International, Palo Alto, California, USA). The primers for GPV were G5-P1 (5′- GAGGTAGACAGCAACAGAAA-3′) and G5-P2 (5′- GCTCGTCCGTGACCATA-3′), and it amplified a 343 bp product. The primers for MDPV were M3-P1 (5’ TAATGGTGGCAGGAATGCACAGTTC-3′) and M3-P2 (5′- TGTTACCATGATGTCTGAAAT-3′), which amplified a 331 bp region. The *T*m values of the two PCR products were 87.5 ± 0.3 °C for GPV and 86.05 ± 0.21 °C for MDPV.

PCR products from GPV and MDPV were cloned into a pMD19-T vector to construct the plasmids pGDA (for GPV) and pMP (for MDPV) using DNA Ligation Kit and *E.coli* Competent Cell JM109 from Takara Biotechnology (Dalian) Co., Ltd.

The Real-time PCR reactions were performed using SYBR® Premix Ex Taq™ (Tli RNaseH Plus) kit (Takara Biotechnology (Dalian) Co., Ltd) with a Mastercycler® ep realplex system (Eppendorf, Germany) to detect SYBR Green. The reaction was conducted in 20 μL volume and the optimized final reaction conditions contained 10 μL SYBR® Premix Ex TaqTM (Tli RNaseH Plus), 0.5 μL each of 10 μM forward and reverse primer for both GPV and MDPV, 1 μL of DNA template (0.2 ~ 20 ng) and 6 μL of deionized water to a final volume of 20 μL. The thermal programme was as follows: denaturation at 95 °C for 5 min, 40 cycles of denaturation at 95 °C for 15 s, annealing at 60 °C for 10 s and extension at 72 °C for 15 s.

**Fig. 1:**
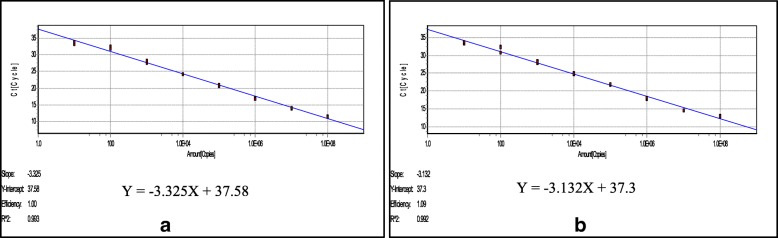
Standard curves of the duplex real-time PCR assay to measure viral copy number of GPV and MDPV. Standard curves for (**a**). GPV and (**b**) MDPV. The x-axis indicates the copy number, ranging from 1 × 10^1^~1 × 10^8^ copies /μL, used in the experiments. The y-axis represents the corresponding cycle threshold (Ct) values. Each data point was amplified in triplicate of each dilution, and the equation below the graph is the standard formulate for regression analysis to calculate viral copy number for (**a**) GPV and (**b**) MDPV

Successive ten-fold dilutions of the standard plasmids pGDA or pMP with copy numbers ranging from 1 × 10^1^~1 × 10^8^ copies/μL were used to construct the standard curve for each parvovirus. Each dilution was tested in triplicates. The standard curve of GPV was linear for 1 × 10^1^~1 × 10^8^ copies/μL, with the correlation coefficient (R2) of 0.993 and efficiency E = 100% (Fig. 1a). The standard curve of MDPV was linear in 1 × 10^1^~1 × 10^8^ copies/μL, with the correlation coefficient (R2) 0.992 and efficiency E = 109% (Fig. 1b). Using this method, we were able to calculate viral copy number using these standard formulas for the regression analysis: Y = − 3.325X + 37.58 (GPV) and Y = − 3.132X + 37.3 (MDPV). This result also indicated that the detection limits of the real-time PCR assay for GPV or MDPV was 10^1^ copies.

**Fig. 2:**
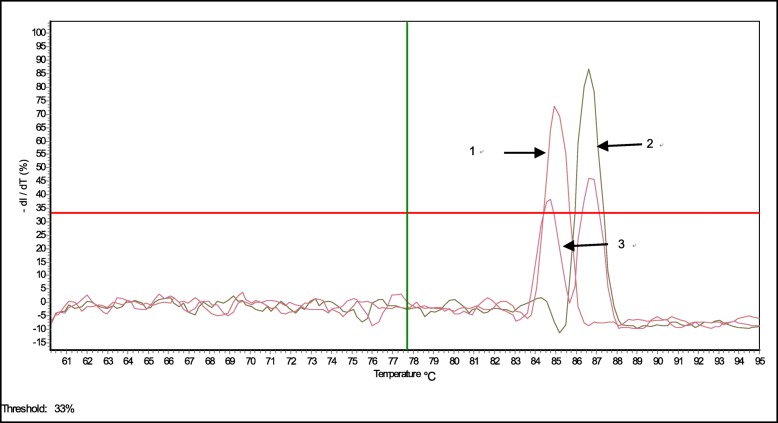
Melting curves of a duplex real-time PCR assay to detect GPV and MDPV. Peaks labeled with the numbers correspond to the viruses as follows: 1, MDPV; 2, GPV; 3, GPV and MDPV. The peaks of melting curves represent the point of inflection of the fluorescence decreasing, which corresponds to the templates that are used in the real-time PCR reactions

After the PCR cycles, a melting curve was generated (with the annealing temperatures 60 °C to 95 °C at a linear transition rate of 0.1 °C/s, where the reaction was held at 16 °C upon completion) to discriminate between the specific amplicons and the non-specific amplification products. The melting peaks were visualised by plotting the negative derivative of the fluorescence intensity over temperature versus the annealing temperature (-dI/dT versus T) produced by the Mastercycler® ep realplex system software 2.0 (Eppendorf, Germany). The *T*m value was defined as the peak of the curve. Single and duplex quantitative PCR were conducted using single or double standard plasmids (pGDA and pMP) described above. The specific single peak of melting curve corresponding to a single GPV template was detected at *T*m = 87.3 ± 0.26 °C, and a specific single peak for MDPV was detected at *T*m = 85.4 ± 0.23 °C. When both viruses were included in one reaction, double peaks for both parvoviruses were detected at *T*m = 87.7 ± 0.24 °C for GPV and *T*m = 84.7 ± 0.28 °C for MDPV (Fig. 2).

**Fig. 3:**
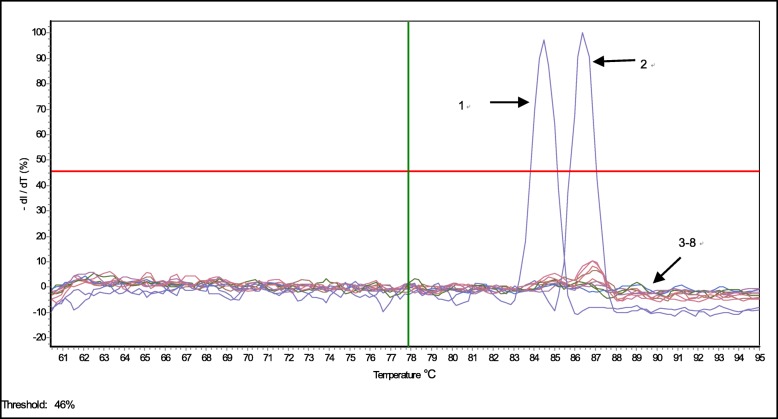
Duplex real-time PCR assay detected GPV and MDPV but not other waterfowl viruses. Fluorescence was detected from (1) MDPV and (2) GPV samples, and fluorescence signal was not observed in the (3–8) control groups, including Novel Duck Reovirus (NDRV), Muscovy Duck Reovirus (MDRV), Duck hepatitis virus (DHV), Duck plague virus (DPV), Goose Paramyxovirus (GPMV) and Egg Drop Syndrome Virus (EDSV) strains

The specificity of the assay was perform using DNA from GPV, MDPV and other waterfowl viruses. Viral DNA was extracted using a QIAamp DNA Mini Kit (QIAGEN) according to manufacturer’s instructions. GPV and MDPV strains, Novel Duck Reovirus (NDRV), Muscovy Duck Reovirus (MDRV), Duck hepatitis virus (DHV), Duck plague virus (DPV) and Goose Paramyxovirus (GPMV) reference strains were obtained from the Laboratory of Animal Viral Diseases at the Institute of Animal Husbandry & Veterinary Medicine, Fujian Academy of Agricultural Sciences, Fujian, China. Egg Drop Syndrome Virus (EDSV) strain was obtained from the China Institute of Veterinary Drug Control. The tested DNA concentrations ranged from 0.3 to 25.5 ng/μL. We did not detect fluorescent signals from other waterfowl viruses (Fig. 3), supporting that the assay is specific to GPV and MDPV.

A series ten-fold dilution of the pGDP and pMP plasmids from 10^6^ to 10^8^ copies/μL was conducted to calculate the coefficients of variation (CVs) for intra- and inter-assay variation in cycle threshold (Ct) values for the real-time PCR assay. The mean intra and inter CV for GPV were 0.87~3.05% and 1.51~3.60%, respectively, and the mean intra and inter CV for MDPV were 0.44~2.21% and 1.29~1.73%, respectively (Table 1).

**Table 1:** Variance analysis of Ct values for real-time PCR assay

Concentration of standard plasmid (DNA copies/μL)	Intra-assay variability			Inter-assay variability		
	CT		CV (%)	CT		CV (%)
	Mean	SD		Mean	SD	
GPV						
1 × 10^8^	10.72	0.17	1.59	10.64	0.16	1.51
1 × 10^7^	14.60	0.45	3.05	14.39	0.32	2.24
1 × 10^6^	17.47	0.15	0.87	17.13	0.62	3.60
MDPV						
1 × 10^8^	13.31	0.12	0.92	13.18	0.19	1.47
1 × 10^7^	16.04	0.35	2.21	15.99	0.21	1.29
1 × 10^6^	18.02	0.08	0.44	17.93	0.31	1.73

To evaluate the application of the assay, a total of 65 artificial infected specimens and 15 field-isolated samples were collected. The artificial infections were conducted in mule ducklings by oral infection with GPV or MDPV strains isolated in the laboratory. The field isolated were collected from suspected cases of GPV or MDPV in goslings and ducklings. Viral DNA were isolated and tested for GPV and MDPV using the duplex real-time PCR assay as described above. The results indicated that 41 of the 65 artificial samples were infected with GPV, which had single peaks in the melting curves at *T*m = 87.3 ± 0.32 °C and the other 24 samples were infected with MDPV, resulting in single melting peaks at *T*m = 85.2 ± 0.26 °C. Among the 15 filed isolated specimens, two were identified as positive for both GPV and MDPV, seven were positive for GPV only, two were positive for MDPV only, and four were negative for either parvovirus. We verified these results using an indirect immunofluorescence assays (IFA) according to the method described by [4]. All the results were consistent between the two methods (data not shown).

Using the Ct value determined from the standard curves, we also demonstrated the viral load of parvoviruses in swab specimens of the cloaca and oropharynx collected from an artificial infection trial conducted in mule ducklings. The dynamic of virus excretion via different routes after oral infection with a short beak and dwarfism syndrome virus (SBDS-GPV) strain [17] was intermittently determined by the real-time PCR assay. Viral DNA was isolated from swabs samples by the DNA extraction kit described above. The viral copy numbers in swab specimens of the cloaca ranged from 10^3.28^ to 10^5.49^ copies/μL at 3–21 days post infection, while the oropharynx swab samples had viral load of 10^3.72^ to 10^5.21^ copies/μL at 3–10 days post infection. For cloaca and oropharynx swabs, viral load reached the highest level at 5 days post infection. Using the IFA method, we only detected the virus at 5–10 days post infection from the cloaca, suggesting that the presented real-time PCR was more sensitive than the serological IFA assay (Table 2).

**Table 2:** Viral load values of SBDS-GPV in cloaca and oropharynx swabs detected by real time PCR assay

Swab Samples	Cloaca	Oropharynx	Cloaca	Oropharynx
Days post infection	Real-time PCR (copies number)		IFA	
3 d	10^4.54^	10^4.64^	0/4	0/4
5 d	10^5.49^	10^5.21^	2/4	0/4
7 d	10^4.17^	10^3.72^	3/4	0/4
10 d	10^4.53^	10^3.74^	1/4	0/4
14 d	10^3.52^	–	0/4	0/4
21 d	10^3.28^	–	0/4	0/4

Despite the prophylaxis taken against waterfowl parvoviruses, parvovirus outbreaks persist in coastal China where vaccination against parvoviruses is widely used in commercial goose and duck flocks [4, 24, 25, 27]. Phylogenetic analysis of VP genes of these newly emerged parvovirus strains indicated that adaptive evolution and genome recombination had occurred due to the accumulation of point mutations and the co-infections among waterfowl parvovirus variants [18, 25, 26]. Therefore, having a fast and precise diagnosis assay for these two parvoviruses is particularly important. In this study, we developed an optimized duplex SYBR Green-I based fluorescence quantitative PCR to detect GPV and MDPV. The primers sequences which were selected in the VP1 sequence of parvovirus based on the compassion of several variants from different subgroups of GPV and MDPV were found to be conserved in spite of those point mutations observed in the last two decades [8, 19, 22, 26]. Therefore, this assay can be used to detect the viruses quickly, and precisely differentiate between GPV and MDPV in one reaction using a melting curves analysis. The method also provides quantitative data that may be used to inform pathogenesis or epidemiological studies, and this was unachievable by conventional PCR or LAMP analyses that have been used until now.

In conclusion, we report a duplex real-time PCR assay that can quantitatively detect and distinguish GPV and MDPV in waterfowl specimens. The assay is rapid, sensitive, specific and reproducible, and will serve as a useful tool for diagnosing, preventing and controlling waterfowl parvovirus infections.

## Abbreviations

Ct: Cycle threshold
CV: Coefficients of variation
DHV: Duck hepatitis virus
DPV: Duck plague virus
EDSV: Egg drop syndrome virus
GPMV: Goose paramyxovirus
GPV: Goose parvovirus
IFA: Indirect immunofluorescence assays
ITR: Inverted terminal repeat
LAMP: Loop-mediated isothermal amplification assay
LPA: Latex particle agglutination test
MDPV: Muscovy duck parvovirus
MDRV: Muscovy duck reovirus
NDRV: Novel duck reovirus
ORF: Open reading frame
PCR: Polymerase chain reaction
SBDS-GPV: Short beak and dwarfism syndrome virus
SN: Seroneutralisation test
